# Von Ziemssen’s Handbook of General Therapeutics

**Published:** 1886-11

**Authors:** 


VON ZIEMSSEN'S HANDBOOK OF GENERAL THERAPEUTICS in seven volumes. Vol. V. General Orthopædics, Gymnastics and Massage. By Professor Dr. Friedrich Busch. Hydro-therapeutics by Dr. W. Winternitz. New York: William Wood & Company, 56 and 58 Lafayette Place. 1886.

The first of the above works meets the varied demands of different classes of our population. Being an elaborate essay rather than a systematic treatise upon orthopædics, the reader must not expect to find in it an exhaustive description of every kind of deformity which orthopædic surgeons have to treat; but it is a valuable work in the estimation of Surgeon Noble Smith, the editor.

In the part devoted to hydro-therapeutics, we are told by the translator, medical officer, F. W. Elsner, that the author has been the modern pioneer, and, conscious of his own merits, has produced a work on hydrotherapy, which for explicitness and scientific character has no superior.

Both on live subjects, treated by masters in their respective departments, affording all the practical knowledge of details that may be desirable for those who are interested in these matters, the book should have a place in every gymnasium and water-cure establishment, as well as in all orthopædic hospitals.

